# Using Mathematical Modeling of Tumor Metabolism to Predict the Magnitude, Composition, and Hypoxic Interactions of Microenvironment Acidosis

**DOI:** 10.1002/bies.70101

**Published:** 2025-12-22

**Authors:** Alzbeta Hulikova, Pawel Swietach

**Affiliations:** ^1^ Department of Physiology, Anatomy & Genetics University of Oxford Oxford UK

## Abstract

In well‐perfused tissues, interstitial composition resembles capillary plasma. Solid tumors break this norm because cancer cell proliferation outpaces vascular expansion, leading to a diffusion‐limited tumor microenvironment (TME) that is notably depleted of oxygen and enriched in acids. The magnitude of tumor acidosis; its chemical composition in terms of [CO_2_] and [HCO_3_^−^] (components of the major extracellular buffer); and its relationship with hypoxia are not intuitive to predict but important to know for designing experiments and contextualising results. We address these timely questions using mathematical models of a monolayer, spheroid, and poorly‐perfused tissue. Our simulations suggest a physiologically realistic TME pH range of 6.7–7.4, reveal a prominence of hypercapnia, and indicate varying levels of HCO_3_^−^ depletion or accumulation arising from fermentation and respiration, respectively. The trajectories of tumor hypoxia and acidosis depend on the balance between aerobic and anaerobic pathways, with important consequences on hypoxic signaling where many responses are pH‐sensitive.

## Graphical abstract

Mathematical model of a perfused cylindrical tumor predicts venous‐end extracellular [O_2_], pH, [CO_2_], and [HCO_3_^−^] in the case of either fermentative or respiratory metabolism, illustrating the relationship between hypoxia and acidosis and the chemical composition of acidosis in the tumor microenvironment.

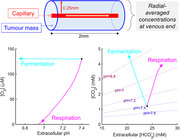

## 1 Introduction

In healthy tissues, short diffusion distances between cells and capillaries collapse solute gradients and stabilize interstitial composition. In contrast, solid tumors are prone to solute heterogeneity because cancer cells divide faster than capillary growth. As a result, the tumor microenvironment (TME) can diverge from plasma composition [1, 2, 3, 4]. Distinct TME features can drive oncogenesis, underscoring the need to elucidate underlying mechanisms and define physiologically realistic solute ranges [5, 6, 7, 8]. A helpful framework for analyzing solute gradients is the diffusion‐reaction model, in which molecules are produced or consumed by chemical reactions, dispersed through the interstitial space by diffusion [9, 10].

The diffusion‐reaction formalism can accurately describe pH and oxygen gradients, two important selection pressures in cancer somatic evolution [11, 12, 13]. The partial pressure of oxygen (pO_2_) spans a range defined by the lower limit of anoxia and the upper limit of arterial normoxia. Within this range, interstitial pO_2_ is set by the balance between vascular supply and mitochondrial consumption. TME pH invariably falls below arterial pH because of metabolic acid production [14, 15, 16], but in the absence of an absolute lower limit for pH, the extent of acidification is difficult to predict and can lead to arbitrary decisions on what pH range to study and attribute to oncogenically significant events. Hence, our first question: *“How low can TME* pH *realistically fall?”* To answer this, we present a quantitative framework that delineates a realistic range for TME pH, and advocate for considering this information in studies of tumor acidosis. This recommendation leads to the second question: *“How should TME* pH *be modeled experimentally?”* H^+^ ions are in dynamic equilibrium with buffers, notably carbonic (CO_2_/HCO_3_^−^). According to acid–base equilibria, pH is determined by the [HCO_3_^−^] to [CO_2_] ratio, so the same pH can arise from many different CO_2_‐HCO_3_^−^ combinations, but not all are physiological. This highlights the importance of constraining [HCO_3_^−^] and [CO_2_] within limits set by tumor metabolism and geometry. CO_2_‐producing respiration also depletes oxygen and establishes a mechanistic coupling between pH and O_2_ gradients over which hypoxic signaling may be influenced by acidosis. This leads to our final question: *“What is a realistic relationship between O_2_ levels and* pH *in the TME?”*

## 2 Closed‐Compartment Model

**FIGURE 1:**
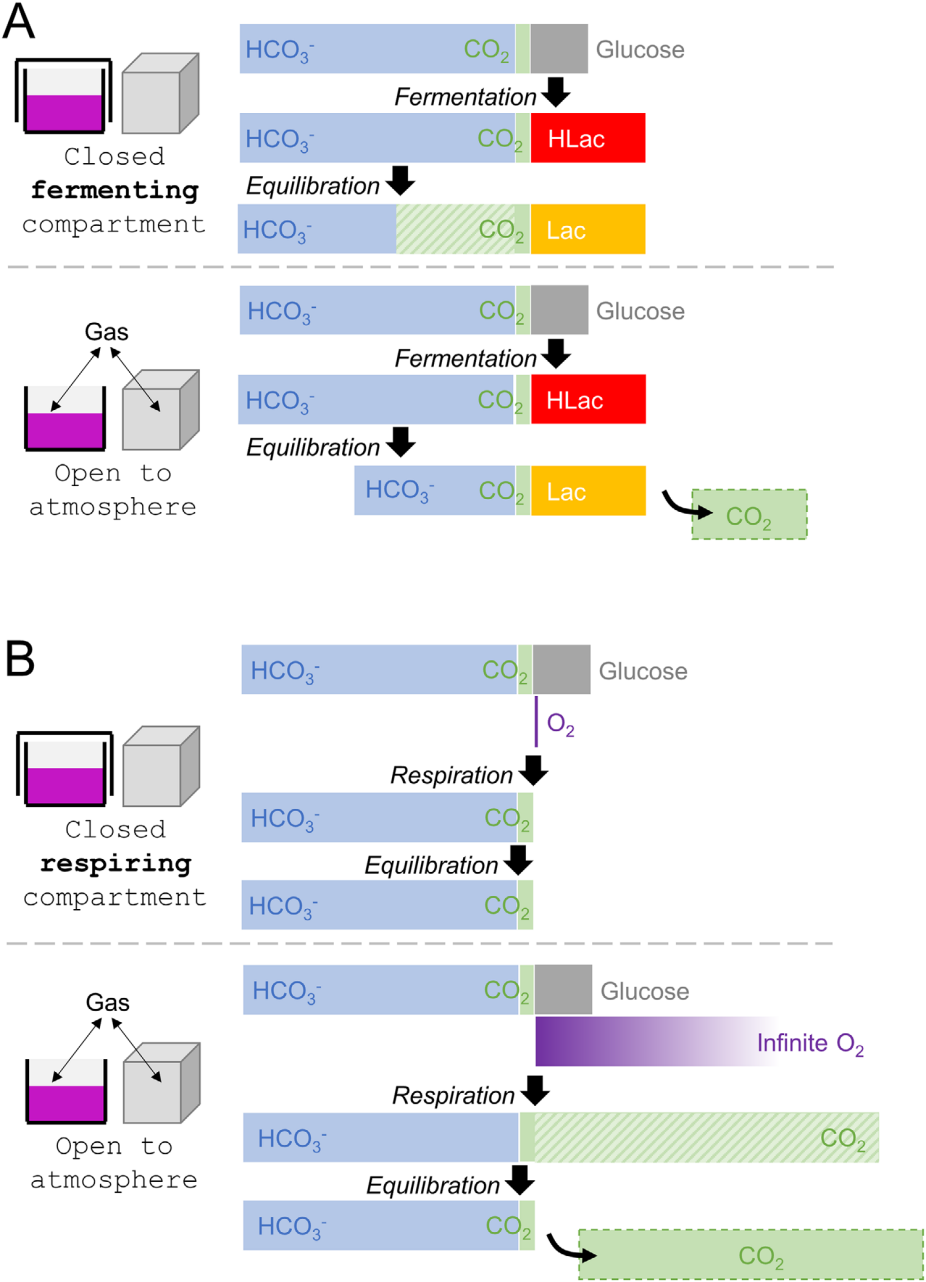
Simple compartment model of the TME. (A) Fermentation in a closed system (e.g., sealed culture dish) results in lactic acid being neutralized by HCO_3_^−^, and conversion to CO_2_ that is retained. Total inorganic carbon (TIC) is constant, but pH decreases significantly. If the system is open for gases (e.g., culture dish in an incubator), excess CO_2_ escapes, but TIC decrease because HCO_3_^−^ was depleted, and pH falls but by less than in a closed system. (B) Respiration in a closed system results in CO_2_ production and a rise in TIC. pH is expected to fall but only marginally because the limiting factor is O_2_ availability. If the system is open for gases, respiration is greatly increased by improved oxygen availability and generated CO_2_ escapes to keep TIC constant. pH is not expected to change.

A starting point is to consider a block of tumor tissue that is abruptly uncoupled from blood flow, allowing metabolic products to accumulate and substrates to deplete. This setting may mimic cultured cancer cells without medium replacement, and is useful for appreciating the extent of pH changes when substrate is allowed to deplete. At 5 mM, the most abundant metabolic substrate is glucose, which can maximally yield 30 mM CO_2_ by respiration or 10 mM lactic acid by fermentation (Figure 1A). Neutralization of lactic acid depletes HCO_3_^−^ and produces an equimolar amount of CO_2_. Reasonably assuming that carbonic buffer is the dominant TME buffer and catalyzed by exofacial carbonic anhydrases [9, 10], equilibrium is estimated by solving the quadratic equation:

$$K=10^{-6.1}=\frac{\left[H^{+}]\times [{\mathrm{HCO}}_{3}^{-}\right]}{\left[CO_{2}\right]}=\frac{\left(10^{-7.4}+x\right)\times \left(0.024-0.01+x\right)}{\left(0.0012+0.01-x\right)}$$

Thus, pH, [HCO_3_^−^] and [CO_2_] could maximally change from the initial 7.4, 24 mM, and 1.2 mM, to 6.2, 14 mM, and 11.2 mM, respectively. The proportionately larger rise in [CO_2_] is because [HCO_3_^−^] depletion is transferred stoichiometrically to a [CO_2_] rise from a lower baseline. The sum of [CO_2_] and [HCO_3_^−^], called total inorganic carbon (TIC), remains constant.

Unlike fermentation, the extent of glucose respiration is limited by oxygen availability (Figure 1B). In the closed‐compartment model, initial pO_2_ would equal arterial 13 kPa, which translates to 130 µM free [O_2_], assuming the hemoglobin‐bound reservoir of ∼9 mM is no longer accessible. At most, this yields 130 µM CO_2_ from 5 mM glucose, a substrate of respiratory quotient (RQ) equal to 1. Thus, TME [CO_2_] is expected to rise from 1.2 to 1.33 mM, a modest 10% increase. Solving the equilibrium equation predicts a 4 nM rise in [H^+^], equivalent to reducing pH from 7.4 to 7.35.

$$K=10^{-6.1}=\frac{\left[H^{+}]\times [{\mathrm{HCO}}_{3}^{-}\right]}{\left[CO_{2}\right]}=\frac{\left(10^{-7.4}+x\right)\times \left(0.024+x\right)}{\left(0.0012+0.00013-x\right)}$$

Whilst didactic, this closed‐compartment model is inadequate because no tumor is entirely hermetic. The model can be modified to allow gas exchange, a scenario resembling cell culture with access to a constant atmosphere (i.e., incubator). CO_2_ produced in excess of 1.2 mM can escape, reducing acidification (Figure 1A,B), and with access to atmospheric oxygen, all glucose could be respired (Figure 1B). This ‘open’ model explains why respiring cells do not acidify media, whereas fermentation leads to progressive acidification and inevitable loss of HCO_3_^−^, and hence of TIC, that must be corrected with medium replacement.

## 3 Toward a Diffusion Model

**FIGURE 2:**
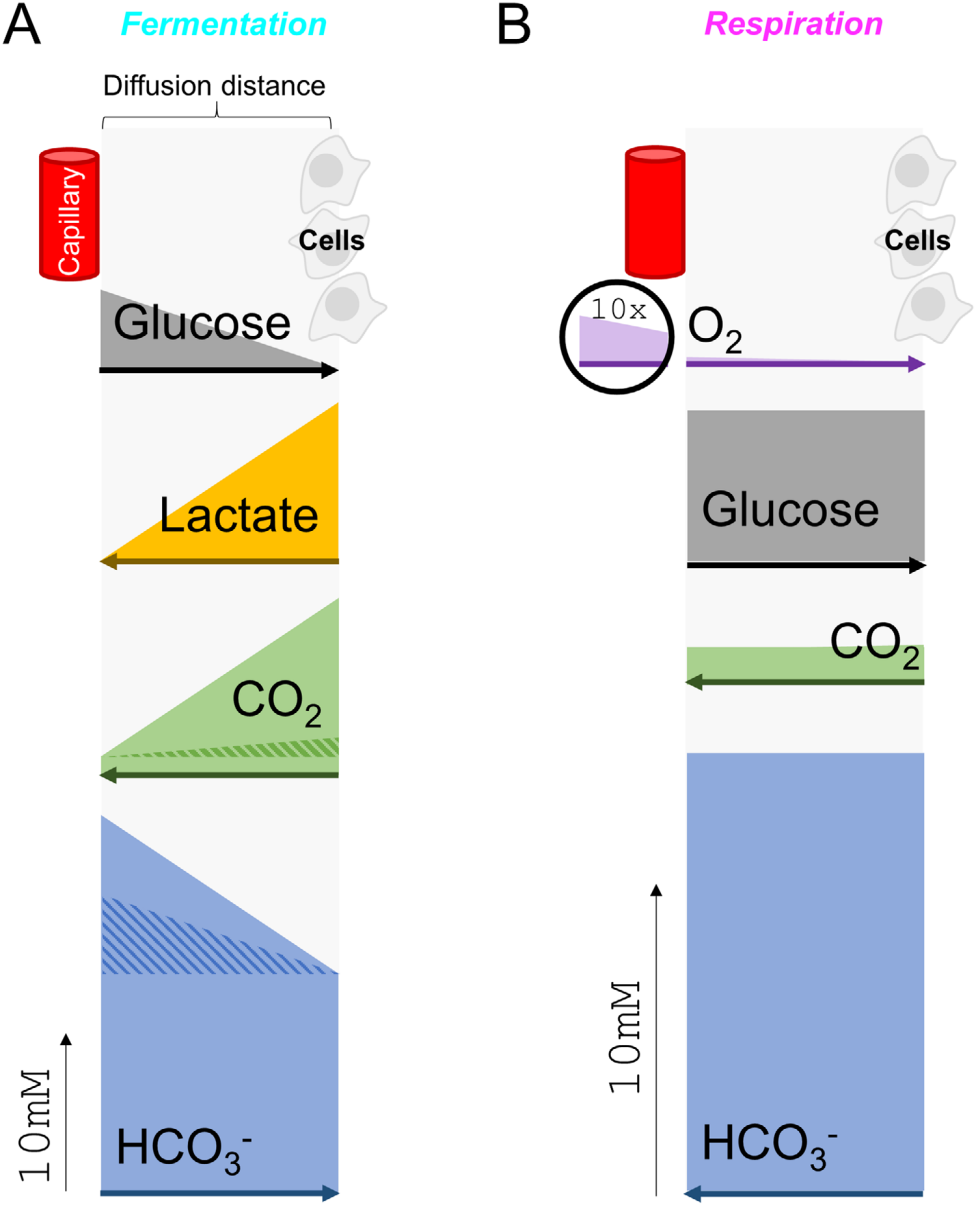
Toward a diffusion model of extracellular solute fluxes in the TME. (A) Cells, separated from a capillary, consuming glucose by fermentation at a rate that produces a maximal gradient. This generates an inverse lactate gradient of double the magnitude, assuming lactate and glucose diffuse at the same rate. To balance fluxes, H^+^ ions produced alongside lactate must be removed by means of a CO_2_/HCO_3_^−^ buffer shuttle, involving HCO_3_^−^ flux toward cells in exchange for a CO_2_ counter‐flux. These CO_2_ and HCO_3_^−^ gradients would be equal to the lactate gradient, if diffusion coefficients were equivalent. However, CO_2_ and HCO_3_^−^ diffuse significantly faster than lactate due to their smaller size, and additionally, CO_2_ can also diffuse through the intracellular compartment, which results in smaller gradients, shown by the shaded area. (B) Consumption of glucose by respiring cells is limited by oxygen availability. This results in smaller CO_2_ and HCO_3_^−^ gradients, both directed toward the capillary.

In a mathematical sense, adding a source of substrate and sink for waste products introduces a boundary condition from which solute gradients emerge. Unlike the closed‐compartment model, a system featuring a constant source of metabolic substrate can attain steady‐state, which can be explored by considering the case of complete glucose consumption. The largest possible [glucose] gradient between blood and cells is 5 mM and generates a flux equal to 5 × *D*, where *D* is glucose diffusivity. At steady‐state, glucose influx is balanced by an efflux of its products, after considering stoichiometry. For fermentation, lactate efflux is 10 × *D*, which is expected to erect a 10 mM [lactate] gradient, if lactate and glucose have equal diffusivity (Figure 2A). H^+^ ions, co‐produced with lactate, must have an equal efflux, which takes the form of HCO_3_^−^ exchanging for CO_2_. The molecular components of carbonic buffer are significantly smaller than glucose, therefore their gradients will be significantly smaller than 10 mM. Additionally, CO_2_ is a gas that freely penetrates the entire tumor volume, whereas HCO_3_^−^ diffusion is restricted to the extracellular space. Using a reasonable estimate of the extracellular volume fraction (v_e_) in tumors of 0.25 [17, 18], CO_2_ diffusivity would be ∼4‐fold faster than HCO_3_^−^ (NB: this difference is likely closer to 5:1 because CO_2_ is a smaller molecule than HCO_3_^−^). Taken together, if HCO_3_^−^ diffuses twice as fast as lactate and CO_2_ diffuses another 4‐fold faster, the predicted radial gradients of [HCO_3_^−^] and [CO_2_] are 5 mM and 1.25 mM, respectively (Figure 2A; shaded). Solving the equilibrium equation:

$$K=10^{-6.1}=\frac{\left[H^{+}]\times [{\mathrm{HCO}}_{3}^{-}\right]}{\left[CO_{2}\right]}=\frac{\left(10^{-7.4}+x\right)\times \left(0.024-0.005+x\right)}{\left(0.0012+0.00125-x\right)}$$

estimates steady‐state pH, HCO_3_^−^, and CO_2_ to be 7.0, 19 mM, and 2.45 mM, respectively, i.e., an acidotic state characterized by reduced [HCO_3_^−^] and raised [CO_2_].

Similar reasoning can be applied to understand how respiration affects pH (Figure 2B). The largest possible O_2_ gradient across the TME is 0.13 mM, and assuming a RQ of 1 and equal O_2_ and CO_2_ diffusivities, the maximal [CO_2_] gradient arising from respiration would be 0.13 mM. This represents a modest respiratory acidosis, when referenced to the CO_2_ baseline of 1.2 mM. There are noteworthy assumptions and limitations. First, the model assumes no O_2_ buffering in the interstitial fluid. Although the most substantive O_2_ buffer, hemoglobin, is confined to blood, leakage from ruptured red cells could increase oxygen flux. Similarly, oxygen binders inside cells could facilitate radial oxygen transfer. These factors could increase the magnitude of respiratory acidosis. Conversely, substrates of lower RQ, such as fatty acids or amino acids, would release less CO_2_. A limitation that applies to both respiratory and fermentative models presented thus far is the lack of spatial considerations, reaction kinetics, or precisely defined diffusion processes. To that end, a more formal diffusion‐reaction model is warranted, and a suitable starting point is a 3‐D spheroid.

## 4 Diffusion‐Reaction Model: Spheroid

**FIGURE 3:**
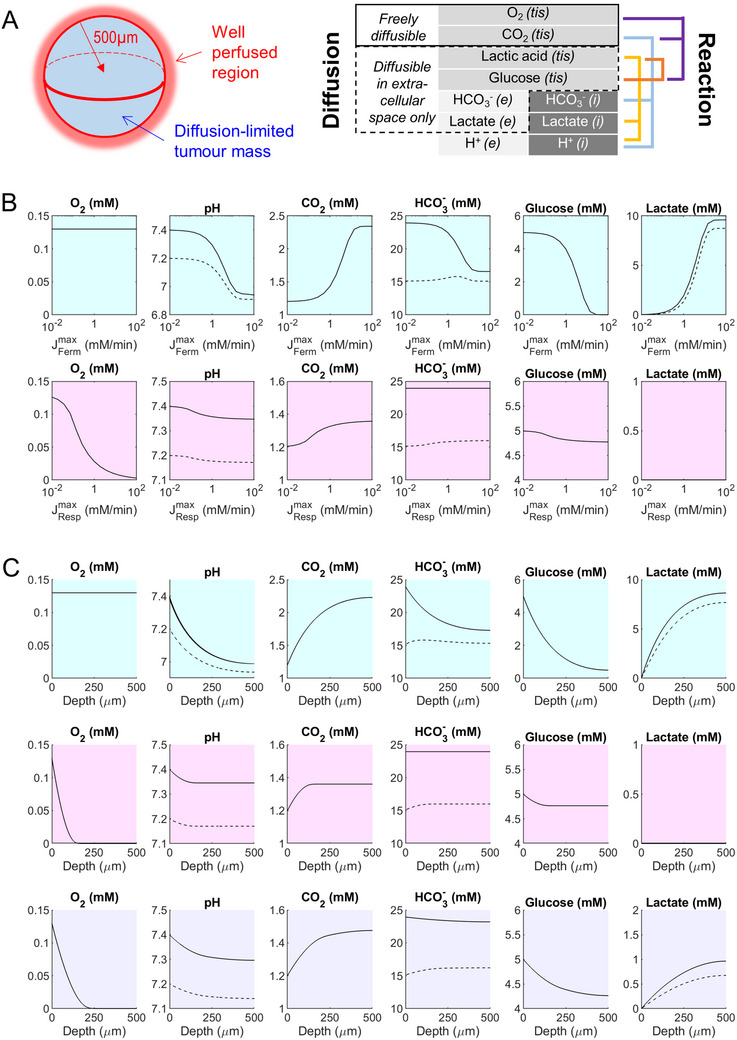
Diffusion‐reaction modeled in spherical geometry. (A) Spheroid of 500 µm radius surrounded by the equivalent of well‐stirred arterial blood. The model consists of solutes, including gases O_2_ and CO_2_ that penetrate intra‐ and extracellular spaces, solutes that permeate membranes by facilitated transport (glucose, lactic acid), and solutes restricted to intra‐ and extracellular spaces (H^+^, HCO_3_^−^, lactate). Reaction terms describe fermentation, respiration, and buffering. CA, carbonic anhydrase; Resp, respiration; Ferm, fermentation. (B) Modeling solute levels at the core of spheroids over a range of maximal fermentative (cyan background) or respiratory (magenta background) rates (J_ferm_^max^, J_resp_^max^). (C) Simulated radial profiles of solutes at steady state for a fermentative spheroid (J_ferm_^max^ = 10 mM/min; cyan background), respiratory spheroid (J_resp_^max^ = 0.67 mM/min; magenta background), and a mixed phenotype (J_ferm_^max^ = 5 mM/min and J_resp_^max^ = 0.33 mM/min; purple background). Dashed lines denote intracellular concentrations; solid lines denote extracellular concentrations, or in the case of permeant species, tissue‐pooled concentrations.

A diffusion‐reaction model was solved over the radial symmetry of a sphere, representing a spheroid of radius 500 µm, of which the extracellular volume fraction (v_e_) was set to 0.25 [17, 18]. The model consisted of 10 solutes in total: four solutes that permeate membranes, either passively (O_2_, CO_2_) or facilitated by transporters (glucose on GLUT or SGLT or lactic acid as H^+^‐lactate on monocarboxylate transporters), and three solutes that partition between intra‐ and extracellular compartments (HCO_3_^−^, H^+^, lactate). For membrane permeable and extracellular solutes, the boundary condition at the surface was that of a constant source of solutes, equivalent to arterial plasma. For intracellular solutes, the boundary condition was zero‐flux, or reflection, meaning that solutes cannot exchange with the surroundings unless they pass through the extracellular space. The components of the model are illustrated in Figure 3A and in the Supplement. To prevent glucose from being depleted (i.e., erroneously attain negative values), fermentative rate was modeled as a Hill‐type equation that is half‐maximal at ∼1 mM [19, 20]. Additionally, an inhibitory effect of intracellular acidosis was modeled with a half‐maximal pK_a_ of 7.1 and cooperativity of 2.25, as determined in a panel of pancreatic cancer cell lines [21].

$${\;J}_{\mathrm{ferm}}=J_{\mathrm{ferm}}^{\max}\times \frac{{\left[\mathrm{Glucose}\right]}_{i}}{{\left[\mathrm{Glucose}\right]}_{i}+K_{\mathrm{Glucose}}}\times \frac{{K_{a}}^{n}}{{\left[H^{+}\right]}_{i}+{K_{a}}^{n}}$$

Respiratory rate also factored a [glucose] dependence, in addition to an oxygen‐dependence characterized by half‐maximal activity at 1 µM [22, 23]:

$${\;J}_{\mathrm{resp}}=J_{\mathrm{resp}}^{\max}\times \frac{{\left[\mathrm{Glucose}\right]}_{i}}{{\left[\mathrm{Glucose}\right]}_{i}+K_{\mathrm{Glucose}}}\times \frac{{\left[O_{2}\right]}_{i}}{{\left[O_{2}\right]}_{i}+K_{O2}}$$

The first set of simulations varied maximal fermentative rate (J_ferm_^max^) or respiratory rate (J_resp_^max^) up to 100 mM/min to investigate predictions for pure metabolic phenotypes (Figure 3B). For the fermentative phenotype, these simulations predicted TME pH reaching ∼6.9 at the spheroid core, with ∼5:1 [HCO_3_^−^] depletion and [CO_2_] accumulation, consistent with the ratio of their diffusivities. In the case of respiration, simulations predicted rapid O_2_ depletion, which limits acidification to ∼0.05 units, and restricts metabolism to the oxygenated outer ring. Radial gradients of solutes were solved for J_ferm_^max^ of 10 mM/min, which produces a near saturating effect on TME pH (Figure 3C). As expected, pHe progressively acidifies with depth, drawing intracellular pH to lower levels. Neutralization of lactic acid by HCO_3_^−^ explains CO_2_ build‐up and HCO_3_^−^ depletion. To model radial gradients of respiring spheroids, J_resp_^max^ was set to 0.67 mM/min, i.e., 15‐fold less than fermentation to account for the ∼15‐fold higher per‐glucose yield of ATP of respiration. The model predicted anoxia beyond a depth of ∼200 µm, hence the rise in CO_2_ remains constant beyond this depth. Since HCO_3_^−^ is not chemically consumed, levels do not fall, unlike the case for fermentation. Since only a fraction of the spheroid can metabolize glucose, overall consumption is small and explains the modest respiratory acidosis of pH 7.35. A third simulation of radial gradients assumed a mixed metabolic phenotype with J_ferm_^max^ of 5 mM/min and J_resp_^max^ of 0.33 mM/min. As expected, intermediate radial gradients were observed, with a deeper oxygenated outer ring, smaller CO_2_ build‐up, and small HCO_3_^−^ depletion.

These simulations are useful for understanding solute gradients in spheroids bathed in the relative constancy of culture medium [24]. The extent of acidification attained by lactic fermentation is maximally pH 6.9, which approaches the prediction of the simplified model shown in Figure 2. Significantly greater acid‐loads could be generated with higher [glucose], as is the case for high‐glucose (25 mM) media. A second significant observation is that restricted oxygen penetration limits respiratory capacity. Indeed, a limitation of the spheroid model is that oxygen supply relies on inward diffusion of dissolved gas. A greater degree of respiratory acidosis could be attained by facilitating O_2_ diffusion using a carrier, such as hemoglobin, but this would require a means of perfusing the spheroid with blood, which is not expected in these non‐vascularized structures. Critically, the carrier would have to selectively bind O_2_ over CO_2_ because a non‐specific facilitation of gas transport would also dissipate CO_2_ (hence pH) gradients. A limitation of the spheroid model is that the surface represents a constant supply/sink for solutes, which effectively clamps concentrations and does not accurately replicate a perfused tissue, where solutes are delivered and removed convectively with the blood flow. A poorly‐perfused tumor is better described as a cylinder of tissue surrounding a capillary with blood flow. To that end, a convection–diffusion‐reaction model is necessary [25].

## 5 Convection–Diffusion‐Reaction Model: Krogh Cylinder

**FIGURE 4:**
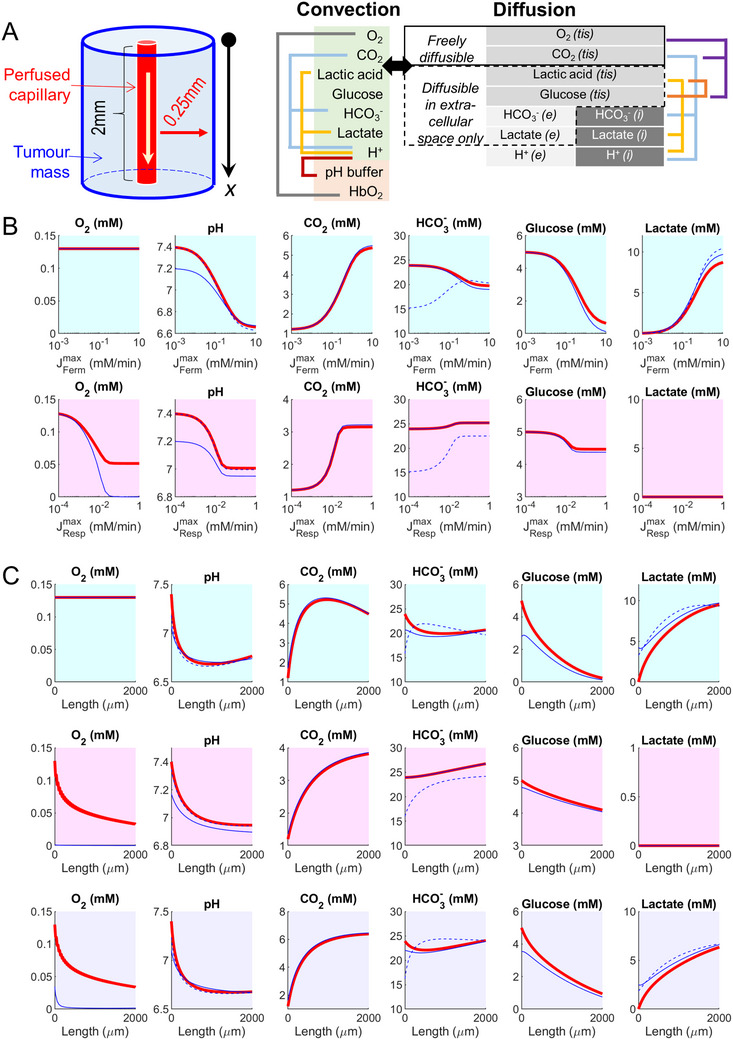
Convection‐diffusion‐reaction modeled in Krogh's cylindrical geometry. (A) Perfused capillary surrounded by a layer of tissue. Blood flow is described by convection of nine solutes, of which seven exchange freely with the tissue and two (pH buffer and O_2_‐Hb) remain confined to blood. In the tissue, the model consists of gases O_2_ and CO_2_ that penetrate intra‐ and extracellular spaces, solutes that exchange between intra‐ and extracellular spaces by facilitated transport (glucose, lactic acid), and solutes restricted to intra‐ and extracellular compartments (H^+^, HCO_3_^−^, lactate). Reaction terms describe fermentation, respiration, and buffering. (B) Modeling solute concentrations over a range of maximal fermentative (cyan background) or respiratory (magenta background) rates (J_ferm_^max^, J_resp_^max^). Tissue concentrations are averaged over the radial distance. Red: blood, blue: tissue. Dashed lines indicate intracellular solutes; solid lines denote extracellular or tissue‐pooled concentrations for permeant solutes. (C) Simulating solute concentration profile along the capillary (red) and tissue (blue) for a fermentative spheroid (J_ferm_^max ^= 10 mM/min; cyan background), respiratory spheroid (J_resp_^max^ = 0.67 mM/min; magenta background), and a mixed phenotype (J_ferm_^max^ = 5 mM/min and J_resp_^max^ = 0.33 mM/min; purple background). Red dashed lines denote intracellular concentrations; solid lines denote extracellular or tissue‐pooled concentrations for permeant solutes.

Perfused tumors can be described as a Krogh cylinder [25] of large radius (Figure 4A). Simulations used a cylinder length of 2 mm, a capillary radius of 5 µm, and a surrounding layer of tissue of thickness 250 µm, which captures the oxygenated rim, as predicted by the spheroid model. Further details are provided in the Supplement. Blood velocity was 1 mm/s, i.e., a transit time of 2 s [26], during which solutes equilibrate between plasma and the TME. Simulations were run for a range of maximal fermentative and respiratory rates (Figure 4B) to interrogate the range of TME pH. When averaged across the length and radius of the tissue, a fermentative Krogh cylinder acidified to pH 6.6, whereas a respiratory phenotype saturated at pH ∼7. Modeling of solute profiles along the length of the tissue was performed for J_resp_^max^ of 0.067 mM/min, i.e., near the saturation point, and a J_ferm_^max^ of 1 mM/min, i.e., 15‐fold higher to account for a ∼15‐fold lower ATP yield per glucose (Figure 4C). Compared to the spheroid model, saturating outcomes were attained at lower metabolic rates because the cylindrical tissue relies on a much smaller capillary volume to supply substrates and remove wastes. At the venous end of a fermentative tissue, blood pH reached ∼6.7, carrying with it ∼9 mM of lactate and ∼4 mM CO_2_, consistent with a near‐complete depletion of glucose (Figure 4B). The model predicts ∼3 mM HCO_3_^−^ depletion and ∼3 mM CO_2_ accumulation, constrained by the constancy of TIC during capillary transit, a transiently closed system. At the venous end of a respiring tissue, glucose consumption is smaller (<1 mM), consistent with rate‐limiting O_2_ penetration. Nonetheless, the Krogh model allows more glucose respiration than the spheroid model because of access to additional O_2_ held on hemoglobin. Still, the entire oxygen content of blood is not sufficient to deplete glucose. Since the Krogh cylinder is a net‐producer of CO_2_, TIC must increase. Metabolism of 1 mM glucose is expected to yield ∼6 mM TIC, split between a ∼2.5 mM rise in [CO_2_] and ∼3.5 mM rise in [HCO_3_^−^], generating an acidification to pH ∼7 (Figure 4C). To model a mixed metabolic phenotype, J_ferm_^max^ and J_resp_^max^ were set to 0.5 mM/min and 0.033 mM/min; this resulted in intermediate spatial profiles and an overall acidification to pH 6.7, significant hypercapnia, and near‐constant [HCO_3_^−^]. The results of these models indicate that a reasonable lower range for TME pHe is ∼6.7, readily explainable by constraints placed on metabolism.

## 6 What Do Simulations Tell us About Tumor Acidosis?

**FIGURE 5:**
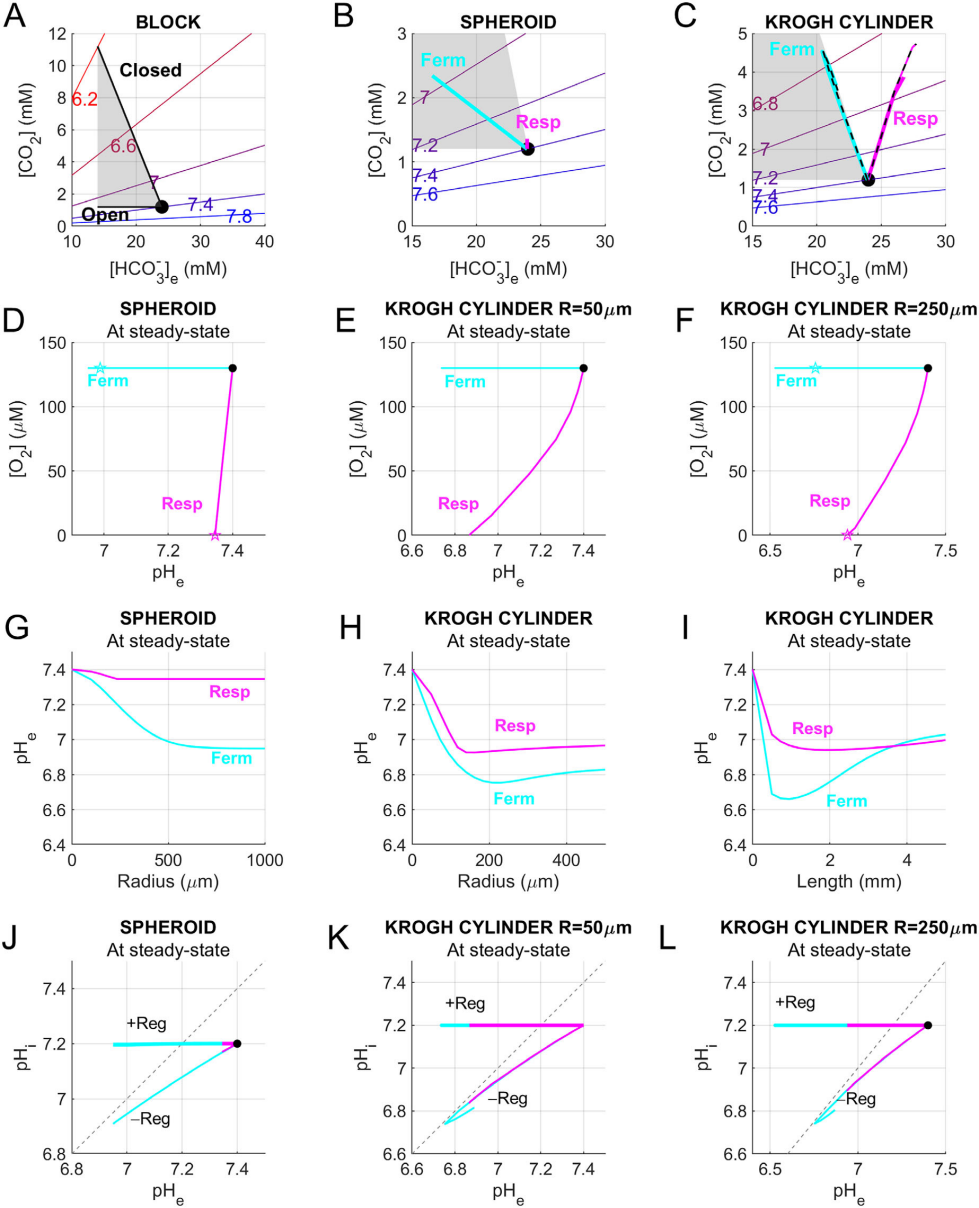
Summary of modeling results. (A–C) Davenport‐like diagrams summarising simulation outcomes. Colored lines (red to blue) denote equilibrium pH for a given CO_2_/HCO_3_^−^ combination. (A). Trajectory of CO_2_/HCO_3_^−^ changes in response to fermentation in a fully closed compartment or a compartment that remains open to gases only. (B) Trajectory of CO_2_/HCO_3_^−^ changes in fermentative or respiratory spheroid. The Shaded area refers to the range defined in (A). (C) Trajectory of CO_2_/HCO_3_^−^ changes at the venous end of the fermentative or respiratory Krogh cylinder of thickness 250 µm or 50 µm (dashed line). (D) Simulated relationship between spheroid radius and pHe for a fermentative or respiratory model (J_ferm_^max^ = 10 mM/min; J_resp_^max^ = 0.67 mM/min). (E) Simulated relationship between Krogh cylinder radius at 2 mm length. (F) Krogh cylinder length at radius 250 µm and pHe for a fermentative or respiratory model (J_ferm_^max^ = 1 mM/min; J_resp_^max^ = 0.067 mM/min). (G) Simulated relationship between extracellular pH (pHe) and intracellular pH (pHi) for models without pH regulation (−Reg) and with pH regulation (+Reg) in a spheroid. (H) Krogh cylinder of radius 50 µm (J_ferm_^max^ = 1 mM/min; J_resp_^max^ = 0.067 mM/min). (I) Krogh cylinder of radius 250 µm (J_ferm_^max^ = 1 mM/min; J_resp_^max^ = 0.067 mM/min). Magenta‐respiration; cyan‐fermentation. (J) Simulated relationship between O_2_ and pHe for fermentative or respiratory spheroids (J_ferm_^max^ = 10 mM/min; J_resp_^max^ = 0.67 mM/min). (K) Krogh cylinder of radius 50 µm (J_ferm_^max ^= 1 mM/min; J_resp_^max^ = 0.067 mM/min). (L) Krogh cylinder of radius 250 µm (J_ferm_^max^ = 1 mM/min; J_resp_^max^ = 0.067 mM/min). Magenta‐respiration; cyan‐fermentation.

Our simulations span scenarios that differ in their physiological relevance and resemblance to experimental conditions. Their predictions can be summarized on a series of Davenport‐like diagrams [27] that plot [CO_2_] against [HCO_3_^−^] over a range of pH (Figure 5A–C). Regular monolayer culture can be modeled as a compartment that is open to gas exchange but closed for non‐volatile solutes. Openness to a normal atmosphere unleashes the full potential of respiration, albeit some pericellular hypoxia may arise in unstirred flasks [28, 29]. This setting may artefactually over‐stimulate respiration beyond the constraints of oxygen delivery in under‐perfused tumors. An open atmosphere also masks respiratory acidosis by allowing CO_2_ to escape. Acidification in such culture systems is driven by fermentation and takes the form of a metabolic acidosis, wherein HCO_3_^−^ is depleted at constant CO_2_ (Figure 5A). At most, pH is expected to fall to ∼7.2 because no more than 10 mM lactic acid could be generated from 5 mM glucose, although a more profound acidosis is possible with supra‐physiological substrate levels, such as 25 mM glucose used in so‐called high‐glucose media.

Good oxygenation and the absence of hypercapnia may resemble cells juxtaposed to capillaries, but this is unlikely to reflect solid tumors because of diffusion barriers. Physiologically, the barriers to oxygen permeation are similar to those obstructing CO_2_ egress; therefore, experimentally replicating the TME ex vivo requires a means of hindering *both* gases. This condition is not met by hypoxic incubation―a routine effort to mimic hypoxia in cultured cells―because O_2_ depletion arises without CO_2_ build‐up. The option of reducing O_2_ and raising CO_2_ in the incubator's atmosphere is challenging to implement because the exact coupling is difficult to predict because it depends on metabolic profile. A practical way of imposing a barrier to gas exchange is to apply a layer of mineral oil atop of the medium [30]. This partial closure allows cells to titrate the degree of hypoxia and hypercapnia as appropriate for their metabolic phenotype. As the barrier increases, the system tends toward a closed state, with two consequences: (i) respiratory suppression from oxygen shortage and (ii) hypercapnia caused by the neutralization of lactic acid with HCO_3_^−^. Depending on the magnitude of the barrier to gas exchange, medium CO_2_, HCO_3_^−^, and pH in most 2‐D culture systems will follow a trajectory nested between the two lines plotted in Figure 5A.

Unlike stirred monolayers, spheroids harbor diffusion distances that can stabilize solute gradients, resembling those of the TME. Spheroids in an open culture system maintain constant [O_2_] and [CO_2_] at their surface, which limits the extent of radial gradients. For a fermentative phenotype, acidosis near the surface will resemble metabolic acidosis, but tends to retain CO_2_ nearer the core. Consequently, the radial profiles of CO_2_, HCO_3_^−^, and pH in the extracellular space of a fermentative spheroid will be nested between a fully‐closed and gas‐open 2‐D culture (gray region in Figure 5B). The metabolism of a purely respiring spheroid is rate‐limited by oxygen penetration, which results in limited CO_2_ build‐up and a resemblance to a fully‐closed system. Since respiration generates TIC, [HCO_3_^−^] increases in tandem with CO_2_, and explains the clockwise deviation from the closed‐compartment (Figure 5B).

In contrast to spheroids, cells in poorly‐perfused tissues exchange solutes with capillary blood, a fluid that is in motion and chemically modified by upstream tissue metabolism. For example, capillary [CO_2_] progressively increases, causing greater CO_2_ retention in downstream tissue. Moreover, blood in a capillary has its TIC trapped during transit, which forces the CO_2_‐HCO_3_^−^ trajectory for fermentative tissues to track closed monolayers, and that of a respiratory phenotype to be further rotated clockwise (Figure 5C). Indeed, an intriguing feature of the visualization shown in Figure 5A–C is that with increasing complexity (monolayer to spheroid to poorly‐perfused tissue), CO_2_/HCO_3_^−^ trajectories rotate clockwise. This transition reflects the retention of CO_2_, hence the emergence of respiratory acidosis. Since respiration (unlike fermentation) always increases TIC, acidosis in respiring tissues is associated with higher [HCO_3_^−^]: not a response that is intuitively linked to acidosis.

The cases modeled in Figure 5A–C had specific geometries, and to investigate a wider range of sizes, simulations were performed for spheroids of radius up to 0.5 mm, Krogh cylinders of radius up to 0.5 mm and length up to 5 mm. For respiratory spheroids, acidification saturation was attained for radius ∼200 µm, which is consistent with the size of the oxygenated outer rim. Acidification in fermentative spheroids saturated at a larger radius of ∼600 µm, which reflects the combination of glucose depletion and glycolytic inhibition at low pH (Figure 5D). For Krogh cylinders, the greatest degree of acidification was attained with radius ∼150–200 µm with both fermentative and respiratory phenotypes and likely reflects an optimal size of the oxygenated rim and acid‐inhibition of glycolysis with greater depth (Figure 5E). Interestingly, tissue length also had an effect on acidification, although the effect was more pronounced with fermentative phenotypes, reaching a pH nadir of 6.7 at a length of ∼1 mm. This effect is likely due to glucose depletion beyond ∼2 mm, which means that downstream tissue is not metabolizing and dilutes the outflow with physiological interstitium (Figure 5F).

Extracellular acidification is ultimately driven by acid‐equivalent fluxes generated by cells. In the models presented thus far, these fluxes represented the metabolic production of CO_2_ by respiration and lactic acid by fermentation. An additional source of acid‐equivalent flux is membrane transport of H^+^ or its chemical equivalents (OH^−^, HCO_3_^−^, and CO_3_^2−^). These fluxes can be readily implemented by coupling equations for intracellular H^+^ and extracellular H^+^ with a Hill‐type equation, such as a representation of Na^+^/H^+^ exchanger (NHE):

$${\;J}_{\mathrm{NHE}}=J_{\mathrm{NHE}}^{\max}\times \frac{{{\left[H^{+}\right]}_{i}}^{2}}{{{\left[H^{+}\right]}_{i}}^{2}+{{\left[K_{NHE}\right]}_{i}}^{2}}$$

Simulations were repeated for NHE featuring K_NHE_ of 10^−6.7^ and J_NHE_^max^ of 10 mM/min, fluxes that are representative of many cancer cells [31]. The effect of implementing a powerful acid‐extruder was visualized in plots of extracellular (pHe) versus intracellular pH (pHi). Without pHi‐regulation, pHi decreased as pHe acidified in spheroids (radius 250 µm) and Krogh cylinders (radii 50 or 250 µm; length 2 mm), with respiration following the same trajectory as fermentation (Figure 5G–I). In the case of Krogh cylinders, the pHi/pHe relationships approach the line of identity and then reverse. The reversal of trajectory is due to depletion of glucose during capillary transit, which means that tissue and blood pH gradually recover as they are diluted by downstream, non‐metabolising tissues. Significantly, the apex of this curve defines the maximal extent of pHe/pHi. When NHE activity is implemented, pHi is homeostatically regulated to the set‐point, here defined as 7.2. Strikingly, the maximal extent of pHe acidification in models with pHi regulation was not substantially affected. This is because regulators like NHE produce a corrective flux only when pHi is acidic; once this is normalized, net flux returns to zero and will not contribute to the steady‐state. Near set‐point pH, the dominant acid‐equivalent flux emitted from cells is carried by CO_2_ and/or lactic acid thus pHi regulators are not expected to make a large contribution. In fact, a significant flux by NHE would cause the cytoplasm to alkalinize to infinity. The reason for the modestly greater pHe drop with NHE for Krogh cylinders of radius 250 µm relates to stimulation of glycolysis at the more alkaline pHi. We conclude that at the steady‐state, pHi regulatory flux will not meaningfully contribute to pHe acidification once pHi is returned to its set‐point.

## 7 Relationship Between Acidosis and Hypoxia

Results of our simulations can be used to explore relationships between oxygen and pH (Figure 5J–L). For purely fermentative phenotypes, acidification is not accompanied by oxygen consumption, irrespective of model complexity. A respiratory phenotype, in contrast, will deplete oxygen and produce acid as CO_2_. For spheroid models, hypoxia is associated with modest acidification because CO_2_ production is limited by O_2_ availability. Total depletion of oxygen is equivalent to a 0.13 mM reduction in [O_2_] and a mere 10% increase in CO_2_ (at least for substrates of RQ of 1), which translates to a ∼0.1 pH unit acidification. A more profound acidification is attainable in the Krogh cylinder model because hemoglobin increases oxygen availability to 9 mM, and capillary CO_2_ can build up in the direction of blood flow. For a respiring Krogh cylinder, CO_2_ production and hence acidification are greater at a radius of 50 µm compared to 250 µm, reflecting the size of the oxygenated outer rim. These O_2_‐pH trajectories demarcate extreme circumstances of a pure fermentative or respiratory phenotype. Since solid tumors are metabolically heterogeneous, any combination of O_2_ and pH lying between the lines marked in Figure 5J–L is plausible. Moreover, changes in oxygen can feedback on metabolism through hypoxic signaling, introducing a dynamic aspect to metabolic heterogeneity. A key transcriptional response to hypoxia is a switch from respiration to fermentation orchestrated by hypoxia inducible factors, most notably HIF1α [32, 33, 34, 35].

**FIGURE 6:**
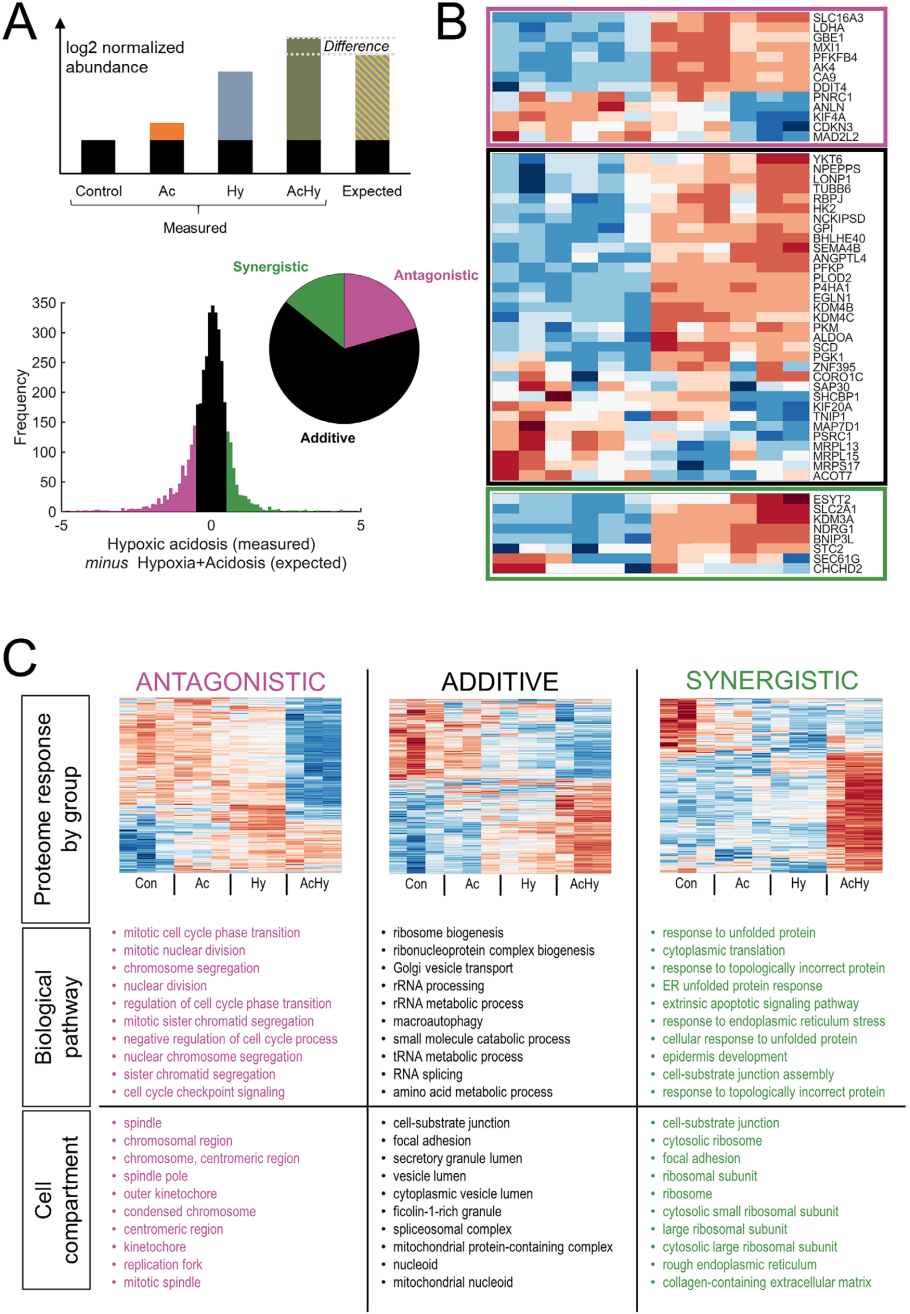
Effect of pH on hypoxic responses. Proteomic analysis (published in White et al) of SW1222 cells after 48 h culture under acidic (pH 6.4), hypoxic (1% O_2_), or their combination. (A) Illustration of the method for quantifying interaction between acidosis and hypoxia. If protein abundance responses to acidosis and hypoxia are additive, then the response to acidotic hypoxia should equal to the sum of its parts. Synergy between hypoxia and acidosis is inferred if the measured response is 0.5 units greater than the expected response. Antagonism is inferred if the measured response is 0.5 units smaller than the expected response. The histogram shows the distribution of this difference across the SW1222 proteome. The pie chart shows the fraction of additive, synergistic, and antagonistic responses. (B) Heatmap of responses in the three groups. (C). Enrichment analysis (ClusterProfiler) for biological pathway and cell compartment of the three groups of proteins, showing the two 10 pathways only.

Compared to alkalotic hypoxia, acidotic hypoxia produces a more transient stabilization of HIF1α, arising from enhanced lysosomal degradation [36] or reduced translation of its mRNA [37, 38]. Consequently, combining hypoxia with acidosis reduces the transcriptional response of at least some hypoxia‐responsive genes [36, 37, 38]. The extent of this interaction can be inferred from a proteomic analysis of cells exposed to acidosis (pH 6.4), hypoxia (1% O_2_), or their combination. SW1222 colorectal cancer cells were selected for this experiment because of their acid resistance, conducive to surviving low pH [36]. After 48 h of treatment, protein abundance was measured and compared across the conditions. If the effects of acidosis and hypoxia were additive, then protein responses determined experimentally under acidotic hypoxia should be equal to the sum of the responses to acidosis and hypoxia presented separately (Figure 6A). However, a difference between the measured and expected response to acidotic hypoxia would signify an interaction which may be synergistic (acidotic hypoxia response greater than the sum of acidic and hypoxia responses) or antagonistic (acidotic hypoxia response smaller than the sum of acidic and hypoxia responses). The distribution of these differences across the proteome is shown in Figure 6A. For log_2_‐transformed abundance, a cut‐off of ±0.5 was used to define the three classes of responses: 2608 additive responses, 822 antagonistic responses, and 569 synergistic responses, alongside 5355 non‐significant responses. Thus, a third of significant responses were not additive, indicating a meaningful interaction between acidosis and hypoxia.

Additive responses to acidosis and hypoxia were broadly associated with protein synthesis and turnover processes—including ribosome and RNP biogenesis, rRNA/tRNA processing, RNA splicing, and vesicle/Golgi trafficking—as well as with macroautophagy and small‐molecule catabolism. Examples of relevant responses are the downregulation of proteins involved in rRNA processing (DDX family, KNOP1, NOPCHAP1, NOP14/16/53, and EXOSC1/8), ribosomal components and maturation factors (RPL members, RIOK1/2/3, BRIX1, and RPF1/2). Altered tRNA metabolism was indicated by changes in aminoacyl‐tRNA synthetases (IARS1/2, LARS1, and MARS2) and TRMT methyltransferases (TRMT61B, 13, 112). RNA splicing was modulated through spliceosomal components (U2AF1/2, SRSF2/10, and PRPF8/40A). Golgi, anterograde and retrograde vesicle trafficking showed upregulation of COPI (COPA/B1/B2/Z1) and COPII (SEC23A/24C/23IP) components, Golgi structural proteins (GOLGA1/5), and regulators of endosomal transport and exocytosis. Metabolic reprogramming encompassed amino acid (GLUD1, GOT2, BCAT2, SHMT2), fatty acid (ACAD8/M/S/VL, ACOT9, ACSL1/4), and carbohydrate (HK1, ENO2, SDHA, SUCLG2) metabolism. The macroautophagy machinery exhibited increased levels of ATG2A/2B/5/7/16L, ULK1/3, and Beclin1, indicating enhanced autophagic flux. These outcomes are expected, as cells must adapt to survive under challenging conditions.

The synergistic effects of acidosis and hypoxia manifested in the induction of stress‐related programs, including ER stress/UPR, responses to misfolded proteins, cytosolic translation, extrinsic apoptosis, and adhesion remodeling. Examples of responses included activation of ER stress and UPR pathways (ATF3, ATF6/6B, ATF7/7B, ERN2, HSPA13, HSP90B1, HSP90AB2B/4B, DNAJA1/C15/C25, and PDI5/6), ribosomal stress (RPS27A/L, EEF2KMT), and extrinsic apoptosis (CASP2, BID, TNFRSF19, TNFAIP8, TP53I11, TP53BP2, TP73, and BCL2L15/L13). Concurrently, protein signatures indicated extracellular matrix remodeling (COL6A1/A3, COL2A1, and ITGB5/6), altered cell adhesion (CEACAM1/5), and epidermal differentiation (multiple KRTs). These patterns align with the cooperative effects of lactic acidosis and hypoxia in the tumor microenvironment and the protective ATF4‐mediated response [37].

In contrast, antagonistic effects of acidosis and hypoxia primarily affected cell‐cycle and mitotic programs, including cyclin‐dependent kinases (CDK1/4/6) and division regulators (CDC20/CDC25B/CDC6/7), as well as DNA replication and repair proteins (MCM2–10, ORC6, CDT2/DTL, BRCA1/2, and RAD51 family), and components of the mitotic spindle and kinetochore machinery (CENP‐E/F, BUB1/BUB1B, KIF15/18B/20A/B/21A/22). This finding indicates that acidotic hypoxia attenuates proliferative programs that are less strongly repressed by either stress alone, and explains the growth arrest and quiescence under acidic hypoxia.

## 8 Conclusions, Recommendations and Limitations

### 8.1 How Low Can TME pH Realistically Fall?

The range of models presented herein—from simple quadratic equations to convection–diffusion‐reaction models of perfused tissue—provide a framework for appreciating the extent of acidification possible in the TME under the constraints of chemistry and physics, but subject to certain assumptions. Although the extreme case of total glucose consumption by fermentation in a closed compartment predicts pH falling to 6.2, this is unlikely to reflect the TME because no tumor is a hermetically closed compartment. This is illustrated by the more modest acidification predicted by the spheroid (“3‐D culture”) and Krogh cylinder (“poorly‐perfused tissue”) models. In the case of total glucose consumption, fermentation is predicted to decrease pH to ∼7, which can be explained in terms of flux‐balance as follows. Complete fermentation drives glucose influx equal to the maximal blood‐tissue difference (5 mM) multiplied by glucose diffusivity. This is counterbalanced by a two‐fold greater lactate flux, reflecting 2:1 stoichiometry, alongside H⁺ ions shuttled by HCO_3_^−^/CO_2_ exchange. Since CO_2_/HCO_3_^−^ diffuses more rapidly than lactate or glucose, the [HCO_3_^−^] gradient and even more so the [CO_2_] gradient are smaller. For a reasonable estimate of CO_2_:HCO_3_^−^:lactate diffusivity of 10:2:1, the Henderson–Hasselbalch equation predicts a pH of ∼7:

$$\mathrm{pH}\;=pK_{a}+\log\frac{\left[{\mathrm{HCO}}_{3}^{-}\right]-2\times \left[\mathrm{Glucose}\right]/2}{\left[CO_{2}\right]+2\times \left[\mathrm{Glucose}\right]/10}$$

This prediction is supported by simulations of spheroids based on a diffusion‐reaction framework. According to the Krogh cylinder model, however, even greater acidifications―down to pH 6.7―are possible, reflecting a more complex relationship between CO_2_/HCO_3_^−^, lactic acid, and lactate once advection is also added to the modeling. Although glucose respiration yields three times as many acid‐equivalents compared to fermentation (6 × CO_2_ versus 2 × lactic acid), the actual yield of acid is restricted by oxygen availability, which is in the sub‐micromolar range, despite high total O_2_ availability in blood (∼9 mM) and rapid gas diffusivity. Consequently, the maximal [O_2_] gradient across the TME is ∼0.13 mM, which is rate‐limiting for respiratory rate. Even if this O_2_ were consumed entirely to produce CO_2_, the effect on pH would be modest on top of the baseline of 1.2 mM [CO_2_].

### 8.2 Limitations of Our Models

Taken together, we propose that the most likely range of TME pH is 6.7–7.4. This range overlaps with measurements of extracellular pH in tumors in vivo [39, 40, 41, 42, 43, 44, 45, 46, 47, 48, 49] with a median of ∼6.8 [50]. Of note, some measurements of TME pH were substantially lower than 6.7, which may suggest additional sources of acidity that our models fail to capture. Indeed, a notable limitation of our simulations is that only glucose metabolism is considered a source of acid. Tumors have access to additional substrates, including blood‐borne non‐esterified fatty acids (NEFA; ∼0.2 mM) [51], lactate (∼1 mM), ketone bodies (<1 mM), or cell‐stored glycogen [52]. Plasma‐borne substrates other than glucose can be metabolized oxidatively and contribute toward hypercapnia and hypoxia. With an average of 17 carbon atoms per molecule, NEFA are the most significant substrates after glucose, but their overall carbon content (17 × 0.2 = 3.4 mM) is ∼10% of the carbon content of glucose (6 × 5 = 30 mM). Thus, a more complete model with all respiratory substrates would have only a small effect on the overall outcomes. We therefore opted for a simplified model featuring glucose metabolism in the interest of didactic accessibility of our models. Unlike plasma‐delivered substrates that are in continuous arterial supply, glycogen is a finite store. At steady‐state, glycogen breakdown must equal production from plasma glucose, which—for the sake of simplicity—could be pooled with the direct route of glucose metabolism via glycolysis. A further source of acidity is the secretion of acid by cancer cells through active transport processes [53], including Na^+^/H^+^ exchangers (NHEs) or V‐type H^+^ ATPases. These fluxes can be substantial but cannot continue perpetually as they are limited by their effect on intracellular alkalinization and inhibition by the ensuing extracellular acidity [31]. Our modeling shows that once pHi is restored, fluxes carried by pHi‐regulators collapse to zero (the definition of attaining a set‐point), which means they cannot meaningfully contribute to extracellular acidification. A perpetual flux by transporters such as NHE would lead to infinite alkalinization of cells, which is not feasible. Expunging cellular content, including acidic organelles, from ruptured cells may lower TME pH instantaneously but this event does not represent a continuous supply of acid, as the case for metabolism. Finally, our models are notable for lacking electrostatic interactions, which may impact the pH distribution [54]. This, however, is poorly defined and therefore difficult to model, but unlikely to be a major contributor to acidification due to short‐circuiting due to abundant ions such as Na^+^ and Cl^−^.

### 8.3 How Should TME pH Be Modeled Experimentally?

Our literature survey of in vitro experiments [37, 51, 55, 56, 57, 58, 59, 60, 61, 62, 63, 64, 65, 66, 67, 68, 69, 70, 71, 72] suggests pH 6.5 as a mean value [50], with some studies–including our own work [73, 74]–investigating responses to pH as low as 6. We advocate for assessing pH‐sensitivity across a broad range, ensuring that the pH range of 6.7–7.4 is adequately interrogated. Experimental control of pH is best exercised by exploiting the prominence of CO_2_/HCO_3_^−^ as the major extracellular buffer, rather than attempting to overcome it using excessive concentrations of exogenous buffers (e.g., HEPES) or entirely replacing this physiological system [75]. In regular monolayer culture, acidosis is most likely attained by fermentation that depletes HCO_3_^−^ whilst [CO_2_] is clamped by the incubator atmosphere. However, pure metabolic acidosis is unlikely in the TME because some retention of CO_2_ is inevitable, as illustrated by the spheroid and Krogh cylinder models. Somewhat surprisingly, [HCO_3_^−^] can fall or rise during acidosis, depending on the modeled geometry and metabolism. For instance, fermentative spheroids tend to deplete [HCO_3_^−^] because TIC cannot increase, whereas respirating Krogh cylinders accumulate [HCO_3_^−^] in tandem with CO_2_ because their TIC must rise. This latter response arises because respiration adds CO_2_ to TIC, which then becomes trapped during capillary transit; consequently, [HCO_3_^−^] increased, but relatively less than the [CO_2_]‐rise, i.e., a net acidosis. We conclude that HCO_3_^−^ depletion is not a universal feature of acidotic TME, even though it is a preferred method for attaining acidosis in cultured media [75]. Pragmatically, it would be impossible to set medium [CO_2_] and [HCO_3_^−^] independently and with adequate precision because metabolic phenotypes are challenging to measure and anticipate. For high‐throughput assays using 96‐well (or similar) plates, and in the interest of batching experiments for better statistical power, it is not practical to vary [CO_2_] for individual experimental conditions. Instead, pH can be controlled robustly by modifying [HCO_3_^−^] through appropriate (osmotically‐compensated) adjustments to medium composition, and we continue to recommend this approach because the alternative of varying [CO_2_] is technologically inaccessible. This recommendation acknowledges that replicating TME acidosis as a metabolic acidosis carries the risk of masking responses to hypercapnia, such as carboxylation [76] or carbamylation [77, 78] which may be particularly important in very acidotic cancers, such as gastric or pancreatic.

### 8.4 What Is a Realistic Relationship Between O_2_ Levels and pH in the TME?

Intuitively, a barrier to oxygen is also a barrier to acid (e.g., CO_2_), therefore, a hypoxic tumor region is also expected to be acidotic. Since the balance between fermentation and respiration can be tuned dynamically through HIF, almost any combination of pH (∼6.7–7.4) and O_2_ (0%–21%) is plausible for the TME in situ. When studying hypoxic signaling, we recommend the inclusion of interactions with acidosis because as many as a third of hypoxic responses can show a synergistic or antagonistic interaction with pH.

## Author Contributions

PS developed, analysed and interpreted the mathematical models. AH analyzed proteomic data and interpreted the hypoxia‐acidosis interactions. PS and AH wrote the manuscript.

## Funding

This work is supported by Bowel Research UK (SG‐24003) and the Medical Research Council (MR/Z506163/1).

## Conflicts of Interest

The authors declare no conflicts of interest.

## Supporting information




**Supporting Information File 1**: bies70101‐sup‐0001‐Appendix.docx.

## Data Availability

Equations for the spheroid and Krogh cylinder models are shown in the Supplement (Appendix). Models presented herein are available for download as stand‐alone executable files at: https://github.com/pawelswietach/Tumour‐pH‐simulations/releases/tag/v1.0.0.
