# Supplementary material for: Using Mathematical Modeling of Tumor Metabolism to Predict the Magnitude, Composition, and Hypoxic Interactions of Microenvironment Acidosis
Source: Bioessays. 2025 Dec 22;48(1):e70101. doi: 10.1002/bies.70101 (PMC12720195; doi:10.1002/bies.70101)
Supplement: Supplementary file 1 — Supporting Information File 1: bies70101‐sup‐0001‐Appendix.docx. [file BIES-48-e70101-s001.docx]

**SPHEROID DIFFUSION-REACTION MODEL:**

Solutes:

1. Tissue O_2_
2. Tissue CO_2_
3. Extracellular HCO_3_^-^
4. Extracellular H^+^
5. Extracellular Lactate
6. Tissue Lactic acid
7. Tissue Glucose
8. Intracellular HCO_3_^-^
9. Intracellular H^+^
10. Intracellular Lactate

Equation:

Solve for radial symmetry (m=2), time t ≥ 0, radial distance r ∈ [0, R]; R = 500 μm

For the i^th^ solute, concentration Uᵢ(r,t) satisfies the partial differential equation:

cᵢ×∂Uᵢ/∂t = (1/r²) ∂/∂r [r²×Dᵢ×∂Uᵢ/∂r] + sᵢ(U,r,t)

Volume fractions:

Intracellular vi = 0.75 and extracellular ve = 0.25.

Coefficients for volume-weighting:

c = [1,1,ve,ve,ve,1,1,vi,vi×(1+30/1000/(2.303×H_i_),vi]

Diffusion coefficients:

D = [2600,2100,1300×ve,0×ve,1000×ve,1000×ve,960×ve,0,0,0] (μm²/s)

Reaction terms: in M/s:

rCO2_e_ = CA×(k_r_×u_3_×u_4_ - k_h_×u_2_)

rCO2_i_ = CA×(k_r_×u_8_×u_9_ - k_h_×u_2_)

rHLac_e_= (k_b_×u_5_×u_4_ - k_f_×u_6_)

rHLac_i_= (k_b_×u_10_×u_9_ - k_f_×u_6_)

J_resp_ = J_resp_^max^×u_7_/(u_7_+10^-3^)×O_2_/(O_2_+10^-6^)

J_ferm_ = J_ferm_^max^×u_7_/(u_7_+10^-3^)×((10^-7.1^)^2.25^)/(u_9_^2.25^+(10^-7.1^)^2.25^)

J_nhe_ = J_nhe_^max^×(u_9_^2^/(u_9_^2^+(10^-6.5^)^2^)-(10^-7.2^)^2^/((10^-7.2^)^2^+(10^-6.5^)^2^))

s_1_ = - 6×vi×J_resp_

s_2_ = 6×vi×J_resp_+ve×rCO2_e_+vi×rCO2_i_

s_3_ = ve×rCO2_e_)

s_4_ = ve×rCO2_e_-rHLac_e_+J_nhe_)

s_5_ = ve×r_HLace)

s_6_ = 2×vi×J_Ferm_+ ve×rHLac_e_+vi×rHLac_i_

s_7_ = -vi×J_Ferm_ + J_Resp_)

s_8_ = vi×rCO2_i_)

s_9_ = vi×rCO2_i_-r_HLaci-Jnhe)

s_10_ = vi×r_HLaci)

Variables:

Carbonic anhydrase activity: CA=100

Hydration rate constant k_h_=0.14 (s^-1^)

Dehydration rate constant k_r_=k_h_×10^6.1^ (M^-1^ s^-1^)

Acid dissociation rate constant k_f_=10^10^ (s^-1^)

Reverse rate constant k_b_=10^13.9^ (M^-1^s^-1^)

Maximal respiratory and fermentative rates J^resp^ and J^ferm^ are set by user

Initial conditions:

Uᵢ(r,0) = [0.13e-3,1.2e-3,24e-3,(1.2/24)×10^-6.1,0,0,5e-3, 15e-3,(1.2/15)×10^-6.1,0](M)

Boundary conditions:

Symmetry at r = 0: dUᵢ/dx(0,t) = 0

Dirichlet at r = R: Uᵢ(R,t) = Uᵢ(r,0)

**KROGH CYLINDER CONVECTION-DIFFUSION-REACTION MODEL:**

Solutes:

1. Blood O_2_
2. Blood CO_2_
3. Blood HCO_3_^-^
4. Blood H^+^
5. Blood Lactate
6. Blood Lactic acid
7. Blood Glucose
8. Blood pH buffer
9. Blood oxyhaemoglobin
10. Tissue O_2_
11. Tissue CO_2_
12. Extracellular HCO_3_^-^
13. Extracellular H^+^
14. Extracellular Lactate
15. Tissue Lactic acid
16. Tissue Glucose
17. Intracellular HCO_3_^-^
18. Intracellular H^+^
19. Intracellular Lactate

Geometry:

Tissue radius R_tis_=250 (μm)

Capillary radius R_cap_=5 (μm)

Capillary and tissue length L=2000 (μm)

Blood velocity v=1000 (μm/s)

Tissue volume fractions:

Intracellular vi = 0.75 and extracellular ve = 0.25.

Equation:

Solve for one-dimensional (m=0) capillary length x ∈ [0, L]; L = 2000 μm, time t ≥ 0

For the i^th^ solute, concentration Uᵢ(r,t) satisfies the partial differential equation:

cᵢ×∂Uᵢ/∂t = ∂/∂r [Dᵢ×∂Uᵢ/∂r] + sᵢ(U,r,t)

Coefficients for volume-weighting:

c = [1,1,1,1,1,1,1,1,1,1,1,ve,ve,ve,1,1,vi,vi×(1+30/1000/(2.303×H_i_),vi]

Diffusion coefficients in tissue extracellular compartment:

D = [0,0,0,0,0,0,0,0,0,2600,2100,1300×ve,0,1000×ve,1000×ve,960×ve,0,0,0] (μm²/s)

Volumetric exchange:

Blood gain from tissue: Q^b^=2×D/(R_cap_×log((R_tis_+R_cap_)/R_cap_))/R_cap_

Tissue gain from blood: Q^t^=2×D/(R_cap_×log((R_tis_+R_cap_)/R_cap_))×R_cap_/((R_cap_+R_tis_)^2^-R_cap_^2^)

Reaction terms: in M/s:

rCO2_b_ = CA_b_×(k_r_×HCO3_b_×H_b_ - k_h_×CO2_b_)

rHLac_b_= (k_b_×Lac_b_×H_b_ - k_f_×HLac_b_)

rHBuf_b_= (k_f,buf_×(TBuf-HBuf_b_)×H_b_ - k_b,buf_×HBuf_b_)

rHbO2_b_= (k_on,Hb_×(THb-O2Hb_b_)×O2_b_ – k_on,Hb_×K_HbO2_(O2_b_)×O2Hb_b_)

rCO2_e_ = CA_t_×(k_r_×HCO3_e_×H_e_ - k_h_×CO2_t_)

rHLac_e_= (k_b_×Lac_e_×H_e_ - k_f_×HLac_t_)

rCO2_i_ = CA_t_×(k_r_×HCO3_i_×H_i_ - k_h_×CO2_t_)

rHLac_i_= (k_b_×Lac_i_×H_i_ - k_f_×HLac_t_)

mmO2 = O2_t_/(O2_t_+10^6^)

mmG = Glu_t_/(Glu_t_+10^3^)

mmH = ((10^-7.1^)^2.25^)/(H_i_^2.25^+(10^-7.1^)^2.25^)

J_Resp_ = J_Resp_^max^×mmG×mmO2

J_Ferm_ = J_Ferm_^max^×mmG×mmH

s_1_ = Q^b^_1_×(u_1_-u_10_) - v×dBdx_1_ - rHbO2_b_

s_2_ = Q^b^_2_×(u_2_-u_11_) - v×dBdx_2_ + rCO2_b_

s_3_ = Q^b^_3_×(u_3_-u_12_) - v×dBdx_3_ - rCO2_b_

s_4_ = Q^b^_4_×(u_4_-u_13_) - v×dBdx_4_ - rCO2_b_ - rHLac_b_ - rHBuf_b_

s_5_ = Q^b^_5_×(u_5_-u_14_) - v×dBdx_5_ - rHLac_b_

s_6_ = Q^b^_6_×(u_6_-u_15_) - v×dBdx_6_ + rHLac_b_

s7 = Q^b^_7_×(u_7_-u_16_) - v×dBdx_7_

s_8_ = - v×dBdx_8_ + rHBuf_b_

s_9_ = - v×dBdx_9_ + rHbO2_b_

s_10_ = Q^t^_1_×(u_10_—u_1_) - 6×vi×J_resp_

s_11_ = Q^t^_2_×(u_11_-u_2_) + 6×vi×J_resp_ + vi×rCO2_i_ + ve×rCO2_e_

s_12_ = Q^t^_3_×(u_12_-u_3_) - ve×rCO2_e_

s_13_ = Q^t^_4_×(u_13_-u_4_) - ve×rCO2_e_ - ve×rHLac_e_ + ve×J_nhe_

s_14_ = Q^t^_5_×(u_14_-u_5_) - ve×rHLac_e_

s_15_ = Q^t^_6_×(u_15_-u_6_) + 2×vi×J_Ferm_ + ve×rHLac_e_ + vi×rHLac_i_

s_16_ = Q^t^_7_×(u_16_-u_7_) - vi×(J_Ferm_ + J_Resp_)

s_17_ = - vi×rCO2_i_

s_18_ = - vi×rCO2_i_ - vi×rHLac_i_ -vi×J_nhe_

s_19_ = - vi×rHLac_i_

Variables:

Half-maximal rate of glucose consumption: K_glu_=1mM

Half-maximal rate of oxygen consumption: K_mit_=1µM

Carbonic anhydrase activity in tissue: CA_tis_=100

Carbonic anhydrase activity in blood: CA_bld_=10^4^

Hydration rate constant: k_h_=0.14 (s^-1^)

Dehydration rate constant: k_r_=k_h_×10^6.1^ (M^-1^ s^-1^)

Acid dissociation rate constant: k_f_=10^10^ (s^-1^)

Reverse rate constant: k_b_=10^13.9^ (M^-1^s^-1^)

Maximal respiratory and fermentative rates J^resp^ and J^ferm^ are set by user

Total non-bicarbonate pH buffering: T_Buf_=45/1000 (M)

Reverse buffering rate constant: k_fbuf_=10^0.2^ (s^-1^)

Forward buffering rate constant: k_bbuf_=10^8^ (M^-1^s^-1^)

Total oxygen binding on haemoglobin: T_Hb_=4×2.33/1000 (M)

On-rate of oxygen binding to haemoglobin: k_onHb_=10^6^ (M^-1^s^-1^)

Initial conditions (M):

Blood: [0.13/1000,1.2/1000,24/1000,(1.2/24)×10^-6.1^,0,0,5/1000,T_Buf_/(1+10^7.4-7.2^),T_HbO2_]

Tissue: [0.13/1000,1.2/1000,24/1000,(1.2/24)×10^-6.1^,0,0,5/1000,15/1000,(1.2/15)×10^-6.1^,0]

Boundary conditions:

Blood (i=1,…9) at start of capillary x=0: Uᵢ(0,t) = Uᵢ(x,0)

Tissue (i=10,…19) at start of capillary x=0: dUᵢ/dx(0,t) = 0

Blood (i=1,…9) at end of capillary x= L: dUᵢ/dx(R,t) = 0
